# Prevalence and Genetic Characteristics of Japanese Encephalitis Virus among Mosquitoes and Pigs in Hunan Province, China from 2019 to 2021

**DOI:** 10.4014/jmb.2207.07068

**Published:** 2022-08-24

**Authors:** Qiwu Tang, Zaofu Deng, Shengguo Tan, Guo Song, Hai Zhang, Lingrui Ge

**Affiliations:** 1 Hunan Biological and Electromechanical Polytechnic, Changsha 410128, P.R. China; 2 Animal Husbandry and Fishery Bureau of Ningyuan, Yongzhou 425000, P.R. China; 3 Animal Epidemic Prevention Station of Xiangxi Autonomous Prefecture, Xiangxi 416000, P.R. China

**Keywords:** Japanese encephalitis virus, epidemiology, pig and mosquito, genetic characteristics, Hunan province

## Abstract

Japanese encephalitis virus (JEV), the causative agent of Japanese encephalitis (JE), is an importantly zoonotic, vector-borne virus widely prevalent in Asia. Although JE has been well controlled in China, its prevalence remains a huge threat to the pig industry as well as human health. Herein, we report on our molecular and serological investigations of JEV among pigs from different regions in Hunan Province of China from 2019 to 2021. Collectively, 19.27% (583/3026, 95% Confidential Interval (CI) 17.86-20.68) of sampled pigs were positive for JEV IgG antibody as revealed by indirect enzyme-linked immunosorbent assay, and the seroprevalence of JEV among pigs was significantly associated with the development stage and breeding scale (*p* < 0.01). Meanwhile, 10.99% (42/382, 95% CI 7.86-14.13) of tissue samples of pigs with suspected clinical symptoms of JE and 23.44% (15/64, 95% CI 13.06-33.82) of mosquito batches were JEV-positive via reverse polymerase chain reaction. In addition, the complete E gene sequences of 14 JEV strains identified in this study were amplified and sequenced. Phylogenetic analysis showed that all 14 JEV strains belonged to genotype I-b and displayed a distinct genetic relationship to the present JEV vaccine strain (SA14-14-2). In conclusion, our results revealed not only the severe prevalence of JEV in Hunan Province, but also that JEV I-b might be the predominant genotype in Hunan Province, suggesting therefore that effective measures for JE control are urgently needed.

## Introduction

Japanese encephalitis virus (JEV), belonging to the genus *Flavivirus* of the family *Flaviviridae*, is a major causative agent leading to viral encephalitis in Asia [1]. Especially, the disease (Japanese encephalitis, JE) caused by JEV has been continuously threatening the pig industry in China [2]. JEV is a positive-sense, single-stranded RNA virus that can infect a variety of animals, including pigs, mosquitoes, dogs, rabbits, horses, etc. [3]. Mosquito species are the primary vectors of JEV, while pigs are the main reservoirs that promote the transmission of JEV from animals to humans [2,4]. The clinical symptoms of JE in pigs are mainly characterized by neurological symptoms in piglets, reproductive disorders in sows, and continuous high fever in fattening pigs [5].

JEV has an RNA genome (~11 kb in length) that encodes only one polyprotein, which can be further cleaved into three structural proteins and seven non-structural proteins [6]. According to the genomic characteristics, JEV strains can be classified into five distinct genotypes (I, II, III, IV, and V). Among these, genotype III has been highly prevalent in past years in China, while current studies confirmed that the occurrences of JE in China were mainly caused by JEV genotype I strains [7, 8].

JEV has been widely prevalent in Chinese pig populations for more than sixty years, making it also a poqkrtential threat to human health [9]. Investigating the prevalence of JEV is essential for establishing effective measures to control this disease, and minimize its risk to public health. However, data on the prevalence and genetic characteristics of JEV in Hunan Province in recent years remain limited. To address this issue, 3,026 pig serum samples and 382 clinical tissue samples were collected to investigate the serological and molecular prevalence of JEV in Hunan Province. Furthermore, the complete E gene sequences of 14 JEV strains obtained here were cloned and analyzed.

## Materials and Methods

### The Study Province

Hunan Province is located in the southwest part of mainland China, between the eastern longitudes of 108°47′-114°15′ and northern latitude of 24°38′-30°08′. Hunan Province covers a total area of 211,800 square kilometers, and in recent years, more than fifty million pigs have been sold annually in the province. A humid continental and subtropical monsoon climate is observed in Hunan Province where various mosquito species (such as *Culex tritaeniorhynchus* and *Aedes albopictus*) are widely distributed.

### Sample Collection

From July to October in 2019, 2020, and 2021, serum samples were collected from 3,026 pigs on 89 pig farms nearly covering the entire regions of Hunan Province in China. All pigs in these sampled farms had not been immunized with the commercial vaccine against JE. According to the breeding scales, ~4%, 2%, and 1% of healthy pigs were randomly selected from each small (< 500 pigs), medium (500~3,000 pigs), and large pig farms (> 3,000 pigs), respectively. Furthermore, field tissue samples (*e.g.*, aborted fetus, brain, spleen, and lymph node) were collected from 382 clinically diseased pigs under suspicion of JE at 102 pig farms. Meanwhile, 8,350 mosquitoes were captured from 64 of 102 pig farms mentioned above and divided into 64 pools according to the farms of collection. All samples were handled with standard processes on ice and sent to Hunan Biological and Electromechanical Polytechnic Institute for further research.

### Serological Analysis

A commercial indirect enzyme-linked immunosorbent assay (ELISA) kit (Wuhan-Keqian Animal Biological Products Co., Ltd., China) was used to screen for the presence of anti-JEV IgG antibodies in each serum sample. The operation procedure was performed according to the manufacturer’s instructions and previous descriptions [10]. Simply, the diluted serum sample (1: 40) was added into the tested wells (in duplicate) and incubated at 37°C for 30 min. After washing the uncombined proteins, secondary antibody binding to the JEV IgG antibody was added in each well and incubated at 37°C for 30 min. Subsequently, (amount) SureBlue (Supplier, USA) was added and incubated at room temperature for 10 min before washing three times. Finally, 0.2 N H_2_SO_4_ was added into each well to terminate the reaction. The presence of JEV antibodies was determined via calculating the optical density (OD) value at 650 nm on a microplate reader. Serum samples with supernatant (sample OD/positive sample OD) ≥ 0.21 were considered positive for anti-JEV antibodies.

### RNA Extraction and RT-PCR Amplification

Total viral RNA was extracted from each clinical sample or mosquito pool using Trizol reagent (Invitrogen, USA). The purified RNA genome was reverse transcribed using a PrimeScript RT Reagent Kit (Thermo Fisher Scientific, USA) following the protocols of the manufacturer. Reverse-transcription (RT)-PCR assay was performed to detect the presence of JEV nucleic acids (targeting the NSP5 gene) with one pair of primers [11]. Subsequently, the positive RT-PCR products yielding the expected DNA bands of nearly 200 bp were visible in 1%agarose gel electrophoresis.

### Amplification, Sequencing, and Genetic Analysis

One pair of primers (JEV-E-F: 5’-TTTAACTGTCTGGGAATGGG-3’; JEV-E-R:’-GGCATGCACATTGGTCGCTAA-3’) were designed to amplify the complete E gene sequences of 14 representative JEV strains from different regions identified in this study. PCR reactions (50 μl) were performed according to a previous study [12]. The positive PCR products were purified, cloned into the pUcm-T vector, sequenced by Tsingke Biological Technology Company (Changsha, China), and submitted to the GenBank database (Table 1).

The genetic characteristics of the E2 sequences of 14 novel JEV strains were compared with the reference JEV strains available in the GenBank database via the DNAStar version 7.0 software. Furthermore, a phylogenetic tree based on the E2 gene was reconstructed by MEGA 7.0 software, using the neighbor-joining (NJ) method with 1,000 bootstrap replicates. The information on the reference JEV strains was shown in Table S1.

### Data Analysis

The prevalence of JEV in pigs or mosquitoes with different risks (*e.g.*, region, year, and season) was analyzed using a Chi-square test in SPSS 20.0 software (IBM, USA). *p* < 0.05 was considered statistically significant. Also, the 95% confidence interval (CI) in each group was analyzed in this study.

## Results

### Seroprevalence of JEV among Pigs in Hunan Province

In this study, the overall seroprevalence of JEV among pigs in Hunan Province was 19.27% (583/3026, 95% CI 17.86-20.68), and the average positive rate of JEV IgG antibodies in pigs in 2019 was significantly higher than those in 2020 and 2021 (*p* < 0.05). The prevalence of JEV infection among pigs of different development stages was shown in Table 2. The highest seroprevalence of JEV was observed in gilts (29.19%, 108/370), followed by sows (27.56%, 226/820), piglets (16.87%, 95/944), fattening pigs (13.35%, 49/367), and nursery pigs (10.06%, 95/944), while the lowest prevalence was in boars (6.06%, 2/33). According to the breeding scale, the seroprevalence of JEV among pigs at small (31.66%, 95% CI 27.49-35.83) and medium-sized (24.08%, 95% CI 21.50-26.67) farms was significantly higher than that at large-scale farms (11.91%, 95% CI 10.27-13.55) (*p* < 0.05).

### Epidemiology of JEV among Tissue Samples and Mosquitoes

Of 382 tissue samples from JE-suspected pigs and 64 pools of mosquitoes collected in this study, the average positive rate of JEV nucleic acids was 10.99% (42/382, 95% CI 7.86-14.13) and 23.44% (15/64, 95% CI 13.06-33.82), respectively. In terms of pigs with different clinical symptoms, the positive rates of JEV infection among aborted fetuses (21.35%, 19/89, 95% CI 12.84-29.86) and piglets with encephalitis (16.67%, 11/66, 95% CI 7.86-25.66) were significantly higher than that of others (5.89%, 12/227, 95% CI 2.83-8.95) (*p* < 0.05). Furthermore, the detection rate of JEV during 2019-2021 varied from 8.13% to 23.88%, with a sudden dropping trend from 2019 to 2020 (Table 3). However, the detection rate of JEV infection in mosquito pools in 2019, 2020, and 2021 was 21.45%(6/28,), 25.0% (4/16), and 25.0% (5/20), respectively.

### Phylogenetic Analysis

**Fig. 1:**
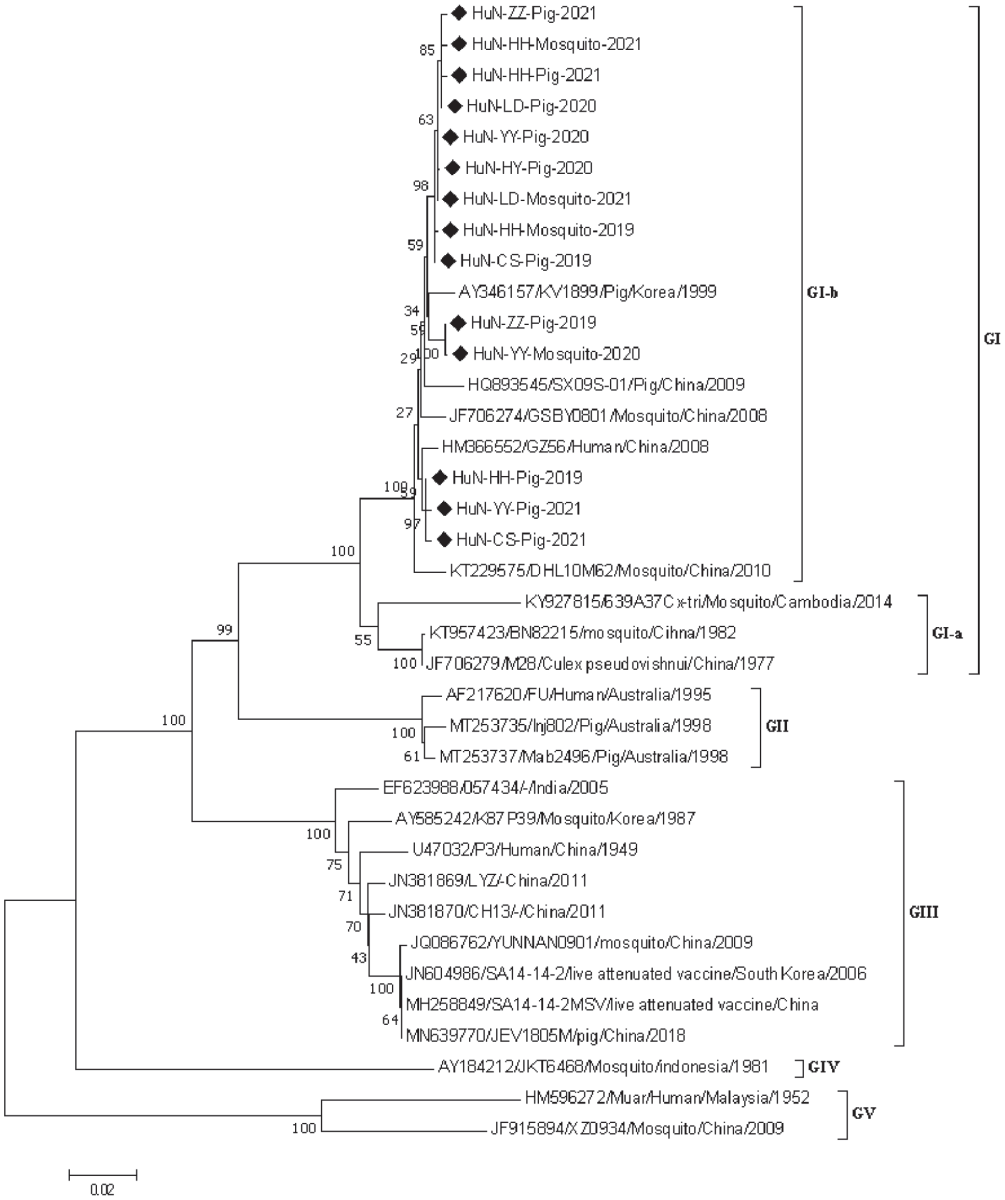
A phylogenetic tree based on the E gene of 14 novel JEV strains and other reference strains was reconstructed by the neighbor-joining method in MEGA 7.0 software [Kimura 2-parameter model; 1,000 bootstrap replicates].

To investigate the evolutionary characteristics of JEV strains identified in this study, the E gene sequences of 14 newly identified JEV strains were successfully amplified and sequenced (Fig. S1). Phylogenetic analysis was performed based on the E gene of 14 newly identified JEV strains and 23 reference strains. In agreement with previous observations [7-8], all JEV strains were classified into five genotypes, and the JEV genotype I group consisted of two clusters (named GI-a and GI-b). All 14 novel JEV strains identified in this study belonged to the genotype GI-b, and 3 of 14 JEV strains were genetically close to the JEV GZ56 strain isolated from human in China (Fig. 1).

### Genetic Characteristics Analysis

The amplified sequences of JEV E gene of the isolates were 1,500 nt in length. They shared 98.8~100.0% identity at the nucleotide sequence level and 98.4~100.0% similarity at the amino acid level. As shown in Table 4, all 14 identified JEV strains shared higher sequence similarity with JEV genotype 1-b strains compared with other genotype strains. Especially, they exhibited low sequence similarity to the JEV live attenuated vaccine strain SA14-14-2 (96.6~97.4% amino acid identity) (Table 4).

Furthermore, amino acid sequence alignment was performed based on the E protein of 14 JEV strains and the live attenuated vaccine strain SA14-14-2 (Fig. S2). There were no amino acid insertions or deletions among these isolates. However, a series of unique amino acid substitutions were observed, which were mainly located in sites 107 (F→L, n=14), 129 (T→M, n=14), 138 (K→E, n=14), 172 (N→I, n=7), 177 (A→T, n=12), 196 (L→Q, n=3), and 222 (A→S, n=10; A→P, n=3; A→L, n=1, etc.).

## Discussion

JEV, which is mainly transmitted by mosquitoes, has been widely prevalent in Chinese pig populations for many years. Moreover, JEV infection leads to neurological disease in humans. Currently, nearly 3 billion people live in JEV-endemic areas and are therefore threatened by JEV infection [8]. Moreover, nearly 68,000 human cases of JE are documented worldwide every year with thousands of deaths, and many survivors may suffer from sequelae, such as dyskinesia and epilepsy [13, 14]. It has been accepted that pigs and mosquitoes play essential roles in JEV transmission from animals to humans, thus obtaining information on the prevalence of JEV among pigs and mosquitoes would be beneficial for the control or eradication of this infectious disease.

The present results showed that the average seropositive rate of JEV IgG antibodies among pigs in Hunan Province was 19.27% (583/3026). This indicates the severe prevalence of JEV in Hunan Province, since these pigs were not immunized with JEV vaccines. Moreover, JEV seroprevalence in pigs in this study was much lower than that in Fujian Province (68.89%) [15], or other regions in China (39.4%) [16], though these investigations were conducted before 2014. Further analysis indicated that the seropositive rate of JEV in Hunan Province in 2019 (23.11%, 190/822) was higher than those in 2020 (17.32%, 209/1207) and 2021 (18.45%, 204/997). A decreased prevalence of JEV in pigs has been observed in China in recent years, which may be due to an improved pathogen detection level as well as that fact that pig farmers have placed greater importance on biosafety, especially after the occurrence of African swine fever in 2018 in China [17].

Moreover, the seroprevalence of JEV infection showed a positive relationship with pig growth and development, excepting piglets and boars, and suggesting that sows and gilts face a higher risk of being infected with JEV. Higher JEV seroprevalence was observed in piglets compared to nursery pigs and fattening pigs, and the maternal antibody from JEV-infected sows and JEV vertical transmission from sows to piglets might be a contributing factor. However, the lowest seroprevalence of JEV in boars was observed, although this could be due to a limited sample size. In addition, the prevalence of mosquito is more rampant at small pig farms because they lack effective biosafety measures, which might be a major factor leading to the higher JEV seroprevalence at small farms (31.66%, 151/477) compared with those at medium (24.08%, 254/1055) and large-scale farms (11.91%, 178/ 1494).

Since JEV cases in humans and pigs usually occur between July to October every year in China, we also performed a molecular epidemiological survey of JEV among pigs suspected of JE and mosquitoes in Hunan Province. The results showed that 42 (10.99%) tissue samples from pigs and 15 (23.44%) mosquito pools were positive for JEV nucleic acids. Positive results in mosquitoes from Hunan Province was higher than that for Zhejiang Province (15.38%, 230/1495) [7], indicating that JEV has been widely prevalent in pigs and mosquitoes in China and this prevalence has varied by region. In addition, more mosquito samples will be collected to further investigate JEV epidemiology since the sample size in this study was limited.

The JEV E protein plays an essential role in various biological processes, including viral entry, host immune response, and viral virulence [16-17]. Moreover, the neutralizing epitopes were mainly located at the domain III of JEV E protein [18], and aa mutations in 337-345 aa, 377-382aa, and 397-403 aa might affect viral immunogenicity [19]. Although a series of amino acid substitutions were observed in the E protein of 14 novel JEV strains compared with the live attenuated vaccine strain SA14-14-2, these substitutions were not located at the major epitope regions of the JEV E domain III [20]. Further experiments will be performed to investigate their effects.

JEV strains currently circulating worldwide are divided into five genotypes, and JEV genotype III strains have been identified in Huaihua City, Hunan Province [21]. However, the phylogenetic analysis showed that all 14 newly identified JEV strains from Hunan Province belonged to the genotype I-b, indicating that JEV genotype I-b strains have been widely prevalent in these investigated regions. Thus, the genetic characteristics of JEV strains prevalent in China should be closely and continuously monitored.

In summary, this is the first study in recent years to comprehensively investigate the prevalence of JEV among pigs and mosquitoes, as well as the genetic characteristics of JEV strains in Hunan Province of China. The results indicated that JEV remains widely prevalent in Hunan Province, and JEV I-b might be the predominant genotype. The data above will provide updated information for a deeper understanding of the molecular epidemiology of JEV in Hunan Province.

## Supplemental Materials

Supplementary data for this paper are available on-line only at http://jmb.or.kr.

## Figures and Tables

**Table 1:** Detailed information of 14 novel JEV strains obtained in the present study.

Strains	Collection year	Isolation region	Pig farm size	Host	GenBank access number
HuN-CS-Pig-2019	2019	Changsha, Hunan	Small	Pig	ON568650
HuN-ZZ-Pig-2019	2019	Zhuzhou, Hunan	Large	Pig	ON568651
HuN-HH-Pig-2019	2019	Huaihua, Hunan	Medium	Pig	ON568652
HuN-HH-Mosquito-2019	2019	Huaihua, Hunan	Small	Mosquito	ON568653
HuN-YY-Pig-2020	2020	Yiyang, Hunan	Medium	Pig	ON568654
HuN-YY-Mosquito-2020	2020	Yiyang, Hunan	Small	Mosquito	ON568655
HuN-HY-Pig-2020	2020	Hengyang, Hunan	Large	Pig	ON568656
HuN-LD-Pig-2020	2020	Loudi, Hunan	Small	Pig	ON568657
HuN-LD-Mosquito-2021	2021	Loudi, Hunan	Large	Mosquito	ON568658
HuN-YY-Pig-2021	2021	Yiyang, Hunan	Large	Pig	ON568659
HuN-ZZ-Pig-2021	2021	Zhuzhou, Hunan	Small	Pig	ON568660
HuN-HH-Pig-2021	2021	Huaihua, Hunan	Large	Pig	ON568661
HuN-HH-Mosquito-2021	2021	Huaihua, Hunan	Medium	Mosquito	ON568662
HuN-CS-Pig-2021	2021	Changsha, Hunan	Small	Pig	ON568663

**Table 2:** Seroprevalence of JEV among pigs in Hunan Province.

Category No. sample	No. positive sample	% (95% CI)	*p*-value
Year	2019 (July-October)	822	190	23.11 (20.12-25.99)	< 0.05
	2020 (July-October)	1207	209	17.32 (15.19-19.45)	Reference
	2021 (July-October)	997	204	18.45 (16.04-20.86)	0.0596
Pig herd	Piglets	492	83	16.87 (13.56-20.18)	0.1027
	Nursery pigs	944	95	10.06 (8.14-11.98)	0.4497
	Fattening pigs	367	49	13.35 (9.87-16.83)	0.2290
	Sow	820	226	27.56 (24.50-30.62)	<0.05
	Gilts	370	108	29.19 (24.56-38.82)	<0.05
	Boars	33	2	6.06 (0-14.20)	Reference
Breeding scale	Large	1494	178	11.91 (10.27-13.55)	Reference
	Medium	1055	254	24.08 (21.50-26.67)	<0.05
	Small	477	151	31.66 (27.49-35.83)	<0.05
Total		3026	583	19.27 (17.86-20.68)	

**Table 3:** The molecular detection rates of JEV among pigs with two risk factors.

	Category	No. sample	No. positive sample	% (95% CI)	*p*-value
Year	2019	67	16	23.88 (16.67-34.09)	< 0.05
	2020	155	13	8.39 (4.03-12.75)	0.9327
	2021	160	13	8.13 (3.90-12.36)	Reference
Clinical symptom	Aborted fetus	89	19	21.35 (12.84-29.86)	< 0.05
	Brain samples in piglets with encephalitis	66	11	16.67 (7.86-25.66)	< 0.05
	Others	227	12	5.89 (2.83-8.95)	Reference
		382	42	10.99	

**Table 4:** Sequence identity in the E gene of JEV strains obtained in this study.

JEV strains compared (GenBank accession no.)	nt/aa level	E
14 JEV strains obtained in this study	nt	98.8~100.0
	aa	98.4~100.0
Compared with the JEV SA14-14-2 live attenuated vaccine strain	nt	87.9~88.1
(JN604986)	aa	96.6~97.4
Compared with the representative JEV genotype I-a strains	nt	93.1~96.2
(KT957423 and KY927815)	aa	97.4~92.2
Compared with the representative JEV genotype I-b strains	nt	98.6~99.4
(HM366552 and JF706274)	aa	99.0~100.0
Compared with the representative JEV genotype II strains	nt	89.1~89.6
(MT253735 and AF217620)	aa	97.6~99.0
Compared with the representative JEV genotype III strains	nt	87.7~88.5
(U47032 and JN381869)	aa	96.8~98.6
Compared with the representative JEV genotype IV strain	nt	81.7~82.0
(AY184212)	aa	94.8~95.2
Compared with the representative JEV genotype V strain	nt	76.1~76.6
(HM596272)	aa	90.8~91.2
