# Supplementary material for: Prevalence and Genetic Characteristics of Japanese Encephalitis Virus among Mosquitoes and Pigs in Hunan Province, China from 2019 to 2021
Source: J Microbiol Biotechnol. 2022 Aug 24;32(9):1120–5. doi: 10.4014/jmb.2207.07068 (PMC9628968; doi:10.4014/jmb.2207.07068)
Supplement: Supplementary file 1 [file jmb-32-9-1120-supple.pdf]

J. Microbiol. Biotechnol.
https://doi.org/10.4014/jmb.2207.07068

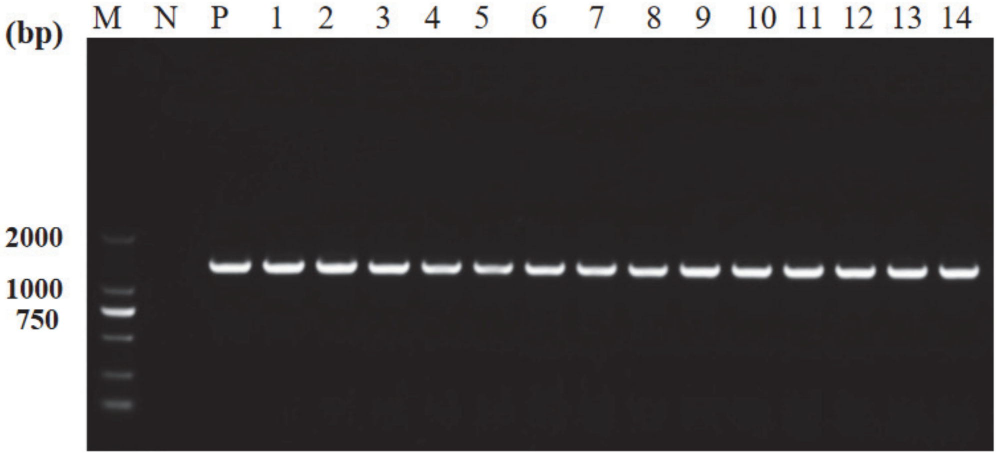

M: DL2000 DNA marker; **N**: Negative control; **P**: Positive control
1-14: 14 JEV-positive samples

Supplementary Figure 2. RT-PCR products of the E gene sequences of 14 novel JEV strains identified in this study.

J. Microbiol. Biotechnol.
https://doi.org/10.4014/jmb.2207.07068

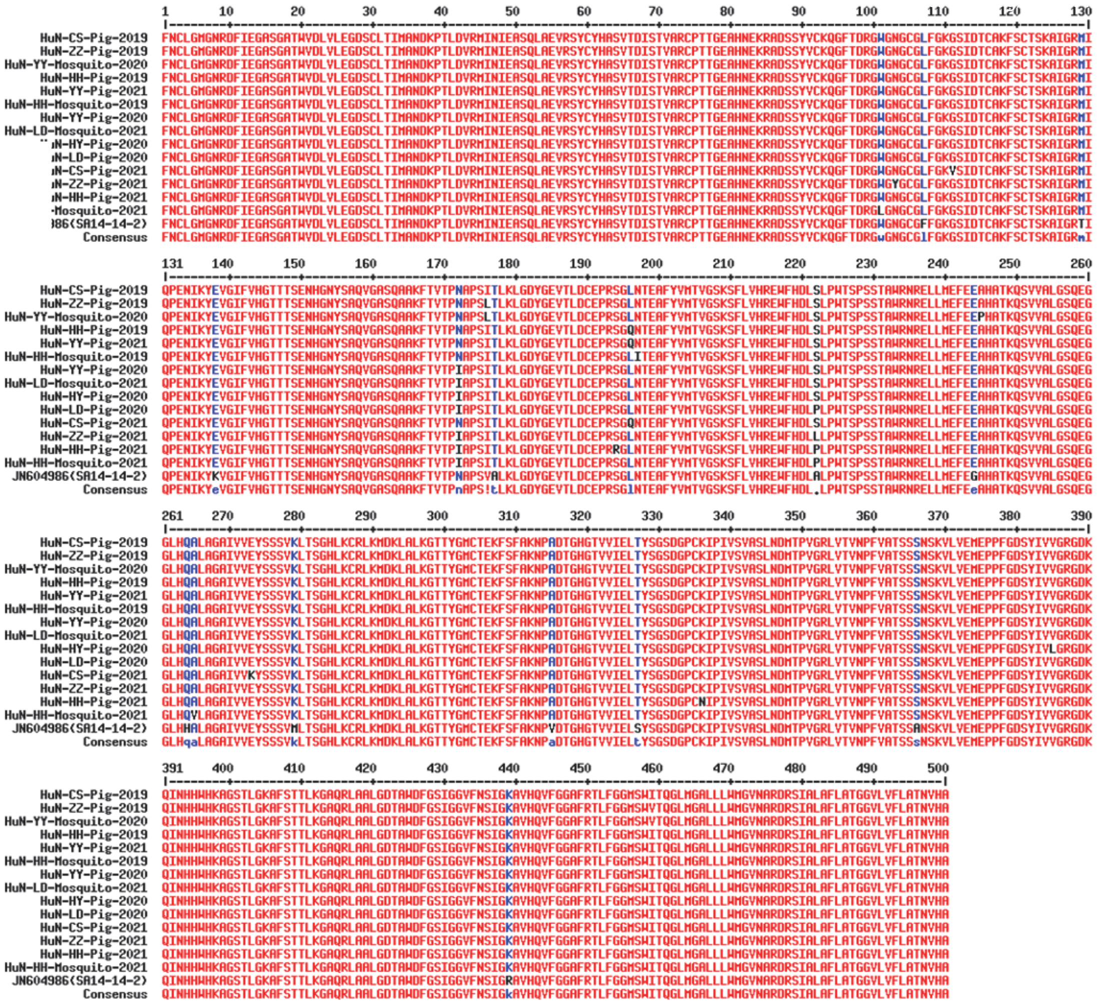

Supplementary Figure 1. Comparison of the E amino acid sequences of 14 newly identified JEV strains with JEV SA14-14-2 strain.

Supplementary Table 1. Detailed information of the reference JEV strains used in the present study.

Strains	Collection year	Isolation region	Host	GenBank access number
M28	1997	China	Culex pseudovishnui	JF706279
BN82215	1982	China	Culex annulus mosquitoes	KT957423
JEV1805M	2018	China	Pig	MN639770
SA14-14-2MSV	-	China	-	MH258849
SA14-14-2	2006	South Korea	-	JN604986
YUNNAN0901	2009	China	Mosquito	JQ086762
CH13	2011	China	-	JN381870
LYZ	2011	China	-	JN381869
639A37Cx-tri	2014	Cambodia	Mosquito	KY927815
KV1899	1999	Korea	Pig	AY346157
GZ56	2008	China	Human	HM366552
DHL10M62	2010	China	Mosquito	KT229575
GSBY0801	2008	China	Mosquito	JF062274
SX09S-01	2009	China	Pig	HQ893545
FU	1995	Australia	Human	AF217620
Mab2496	1998	Australia	Pig	MT253737
Inj802	1998	Australia	Pig	MT253735
K87P39	1987	Korea	Mosquito	AY585242
057434	2005	India	-	EF623988
P3	1949	China	Human	U47032
XZ0934	2009	China	Mosquito	JF915894
Muar	1952	Malaysia	Human	HM596272
JKT6468	1981	Indonesia	Mosquito	AY184212
